# Lack of efficacy of ergocalciferol repletion

**DOI:** 10.3402/jchimp.v2i1.10494

**Published:** 2012-04-30

**Authors:** Amal Kebede, Corey Ephrussi, Meredith Lamanna, Jorge Scheirer, Richard Alweis, Thomas Wasser

**Affiliations:** 1 The Reading Hospital and Medical Center, Department of Medicine, West Reading, PA, USA; 2 Philadelphia College of Osteopathic Medicine, Philadelphia, PA, USA; 3 Jefferson Medical College, Philadelphia, PA, USA; 4 Consult-Stat: Complete Statistical Services, Macungie, PA, USA

**Keywords:** hypovitaminosis D, vitamin D deficiency, ergocalciferol

## Abstract

**Introduction:**

Vitamin D has become an area of intensive scrutiny, both in medical and lay literature. However, there are limited data to suggest proper repletion regimens for those patients who have hypovitaminosis D. Consequently, various methods are used in clinical practice. The aim of this study was to assess the efficacy of various treatment strategies for hypovitaminosis D in an ambulatory internal medicine practice.

**Methods:**

A retrospective chart review between October 2005 and June 2010 of a suburban internal medicine practice was performed via query of the electronic medical record (Centricity, General Electric Healthcare, UK). Patients with a 25-hydroxyvitamin D concentration less than 32 mg/dl were identified and treated. Treatment success was defined as 25-hydroxyvitamin D concentrations greater than 32 mg/dl. Statistical analysis to assess changes in vitamin D level controlling for season, comorbidities, and demographics were used.

**Results:**

A total of 607 treatment episodes were identified, with 395 excluded due to lack of follow-up vitamin D level within 16 weeks, no treatment documented, topical treatment, doxercalciferol treatment, or non-compliance. Of the remaining patients, there were 212 treatment instances on 178 patients. Ergocalciferol 50,000 international units (IU) was used most frequently (71.4% of the time.). A higher initial vitamin D level was positively associated with treatment success (adjusted odds ratio = 1.11, *p*=0.002). Increased doses of ergocalciferol increased the likelihood of treatment success (*p*=0.0011). Seasonal variation was related to posttreatment 25-hydroxyvitamin D concentration as was body mass index (BMI) (*p*=0.003 and *p*=0.044).

**Conclusion:**

Pretreatment levels of 25-hydroxyvitamin D, BMI, season, and vitamin D dose are predictors of successful hypovitaminosis D treatment. Our data suggest that patients with initial 25-hydroxyvitamin D concentrations of <20 should be treated with a higher total dose of ergocalciferol than 50,000 IU for 8 weeks. Further studies, including prospective, randomized trials, are needed to determine an optimal treatment protocol to account for the numerous variables.

The lifetime incidence of hypovitaminosis D in the United States is estimated to be 60% (1). Vitamin D deficiency has been shown to be detrimental in bone health, calcium regulation, and may lead to proximal muscle weakness increasing the risk of falls in the elderly (2). Recent literature has also shown a wide variety of autocrine and paracrine effects of vitamin D, as well as links to increased prevalence of autoimmune diseases and potentially to cancer risk (3–5). Subsequent to literature reviews by Holick, many practitioners use ergocalciferol 50,000 international units (IU) weekly for 8 weeks to treat hypovitaminosis D. If 25-hydroxyvitamin D concentration was still less than 30 mg/dL, another round of 8 weeks of treatment was recommended (1). Recently, the Institute of Medicine (IOM) determined that the average North American requires 600 IU of vitamin D for most adults and 800 IU for patients of age 70 and older (6). In addition, a recent guideline published by the Endocrine Society has provided guidance for evaluation and treatment of vitamin D deficiency (7). This has not received universal acceptance, and there continues to be confusion about the proper treatment regimen, as these most recent guidelines continue to suffer from a lack of support from randomized clinical trials. The aim of this study was to assess the efficacy of various treatment strategies for hypovitaminosis D in an ambulatory internal medicine practice.

## Materials and methods

The setting for this retrospective chart review was a suburban internal medicine ambulatory faculty practice in West Reading, PA (latitude 40° 20′ 8′′ N). Through query of the practice's Centricity electronic medical record (EMR) database (General Electric Healthcare, UK), all patients with 25-hydroxyvitamin D concentrations of < 32 ng/mL between October 2005 and June 2010 were identified. We defined success of vitamin D treatment as a post 25-hydroxyvitamin D concentration of ≥32 ng/mL, which is designated as the lower limit of normal by our laboratory. Compliance with therapeutic delivery was not possible to assess in this retrospective study.

Patients were tested to determine a baseline 25-hydroxyvitamin D concentration with a plan to measure a follow-up of 25-hydroxyvitamin D concentration within 16 weeks of start of a repletion protocol. A duration of 16 weeks was selected because most studies that used retrospective reviews used 8 to 12 week follow-ups, and we felt that a duration of 16 weeks was a reasonable time to allow patients to get posttreatment blood work to maximize sample size but without allowing time for 25-hydroxyvitamin D concentration to deplete again.

### Statistical analysis

Demographic variables were reported to describe the sample. Continuous variables of age, body mass index (BMI), creatinine clearance, and initial Vitamin D level are reported as mean±standard deviation; all other variables were discrete and are reported as count and percent of total.

A gamma statistic, used for ordinal data, compared initial vitamin D level as measured ≤15, 16–20, 21–25, 26–31 against a final determination of therapeutic level after therapy for both individual first treatment patients as well as all treatment episodes.

A linear mixed model, using both pre- and posttreatment levels of vitamin D, was used to assess the change in vitamin D levels, controlling for season and conditions known to impact vitamin D (e.g., obesity, chronic corticosteroid usage, chronic anticonvulsant therapy, malabsorption syndromes). The results were reported in terms of the model's overall significance as well as univariate results. A logistic regression model was used to assess the impact of potential association of comorbidities and demographics with success of vitamin D treatment, and to calculate adjusted odds ratios (OR) for the treatment and associated variables. Standard outcome from a logistic regression includes the Wald statistic for each variable in the model, and the *p*-value is related to each Wald value for all variables in the model, both for the patients’ first treatment (*n*=178) and the total treatment episodes (*n*=212).

## Results

**Fig. 1:**
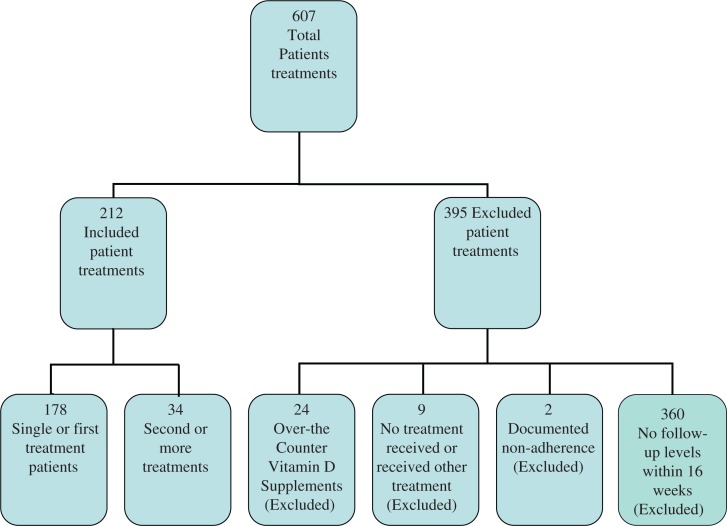
Patient population.

A total of 607 treatment episodes were identified with hypovitaminosis D, of which 73% were female. Of these episodes, 395 were excluded secondary to: lack of follow-up of vitamin D level within 16 weeks, no treatment documented, topical treatment, doxercalciferol treatment, or documented non-compliance (Fig. 1). The study population (Table 1) included 178 patients with a mean age of 61.6 years (±14.1), and 137 (77%) female, who received 212 episodes of vitamin D repletion. Mean initial 25-hydroxyvitamin D concentration was 20.50 ng/mL (±6.24ng/mL) and mean follow-up level was 35.19 ng/mL (±11.92ng/mL). Initial vitamin D level was positively associated with treatment success (Adjusted OR = 1.11, *p*=0.002). In treatment courses using the 8-week ergocalciferol regimen, initial 25-hydroxyvitamin D concentrations of ≤15, 16–20, 21–25, 26–31 ng/mL were associated with significantly different treatment success rates of 60.1, 53.5, 63.0, and 82.9%, respectively (gamma = 0.237 *p*=0.032). Data for the individual first therapy (*n*=178) and the total treatment episodes break down by these groupings can be found in Table 2. The 8-week regimen of 50,000 IU weekly had an overall success rate of 59.3%. Treatment options that provided > 600,000 IU of ergocalciferol were successful in repleting vitamin D deficiency in 76.5% of cases (*p*=0.0011). This shows that higher total doses of supplementation were associated with higher likelihood of achieving normal levels. Seasonal variations were significantly related to posttreatment 25-hydroxyvitamin D concentrations (*p*=0.003). Logistic regression found that BMI was inversely related to vitamin D repletion success for the patients’ first episode of therapy, based on 178 treatment episodes (*p*=0.042) but for analysis on all treatment episodes (*n*=212), there were no significant predictors of vitamin D repletion success and BMI failed to achieve significance (*p*=0.164). Wald's statistics are presented with *p*-values in Table 3. The mean BMI of the individual patient population was 31.2 (±8.3) kg/m^2^.

**Table 1:** Demographic characteristics of the sample (*n*=178) single-treatment patients

Continuous variables	Mean	Standard deviation
Age	61.6	14.1
BMI	31.2	8.3
Baseline vitamin D level	19.9	5.9
Creatinine clearance	85.9	21.2
Gender	Female: 137 (77%)	Male: 41 (23%)
Discrete variables	Yes (%)	No (%)
African-American	2 (1.1)	176 (98.9)
Nephrotic syndrome	0 (0)	178 (100)
Chronic liver disease	2 (1.1)	176 (98.9)
Hyperparathyroidism	5 (2.8)	173 (97.2)
Bariatric surgery	2 (1.1)	176 (98.9)
Malabsorption syndromes	12 (6.7)	166 (93.3)
Chronic anticonvulsants	12 (6.7)	166 (93.3)
Chronic corticosteroids	18 (10.1)	160 (89.9)
HAART	0 (0)	178 (100)
Anti-rejection medications	19 (10.7)	159 (89.3)
Medications that reduce cholesterol absorption	3 (1.7)	175 (98.3)
MVI with D and D	36 (20.2)	142 (79.8)

**Table 2:** Treatment success in patients treated with ergocalciferol 50,000 IU weekly for 8 weeks based on initial 25-hydroxyvitamin D concentration

	Initial therapy (*n*=178)		Treatment episode (*n*=212)	
		
Initial 25-hydroxyvitamin D concentration	Number (%) of patients who had successful repletion	Number of patients in category	Number (%) of patients who had successful repletion	Number of patients in category
≤ 15	28 (60.1)	46	31 (58.5)	53
16–20	23 (53.5)	43	28 (53.8)	52
21–25	34 (63.0)	54	40 (64.5)	62
26–31	29 (82.9)	35	35 (77.7)	45
Total	114 (64.0)	178	134 (63.2)	212
Significance	Gamma = 0.237, *p*=0.032		Gamma = 0.229, *p*=0.024	

**Table 3:** Results of the logistic regression for both first-time treatment per patient (*n*=178) and total treatment episodes (*n*=212) against vitamin D repletion success

	First-time treatment (*n*=178)		Total treatment episodes (*n*=212)	
		
Variable	Wald	*p*-value	Wald	*p*-value
Age	0.115	0.734	0.641	0.423
Gender	0.359	0.549	0.168	0.682
BMI	4.126	0.042	1.938	0.164
Creatinine clearance	1.063	0.302	2.445	0.118
Chronic liver disease	0.000	0.999	0.001	0.980
Hyperparathyroidism	0.375	0.540	0.036	0.850
Bariatric surgery	0.617	0.432	0.645	0.422
Malabsorption syndromes	0.012	0.914	0.019	0.891
Chronic anticonvulsants	0.038	0.845	0.045	0.832
Chronic corticosteroids	0.081	0.775	0.067	0.796
Anti-rejection medications	0.676	0.411	0.319	0.572
Medications that reduce cholesterol absorption	0.001	0.975	0.000	0.989
MVI with D and D	1.227	0.268	1.998	0.158

## Discussion

Treatment duration and dose for hypovitaminosis D are still not standardized. Our study shows that patients treated with ergocalciferol 50,000 IU for 8 weeks achieved treatment success in less than 60% of treatment instances. Success rates varied depending on initial 25-hydroxyvitamin D concentration. Patients with lower initial levels were far less likely to achieve treatment success. Similarly, patients treated with higher total dose of ergocalciferol and lower BMI were more likely to achieve treatment success. This echoes the findings of a recent large Veterans Administration study that once again showed that baseline BMI, ethnicity, and total dosage were significant factors in predicting vitamin D insufficiency and effectiveness of repletion with standard dosing regimens recommended by the IOM (8). The authors concluded on the basis of their study that the IOM report underrepresented the true prevalence of vitamin D insufficiency in the United States as well as failed to take into account the role of ethnicity, BMI, and other individual factors that make a ‘one size fits all’ approach, as previously posited by Holick, and codified by the IOM, as inappropriate (8). However, the study did not assess baseline 25-hydroxyvitamin D concentrations as a predictor for treatment success. Interestingly, our study did confirm that there was a season-affected posttreatment 25-hydroxyvitamin D concentration. Winter was associated with higher posttreatment levels as opposed to autumn, perhaps because of unmonitored factors such as higher proportion of patients than anticipated who might vacate to or live in more seasonal areas in the wintertime, which are also included in the study population. Overall, the general practitioner is left with a number of risk factors, which implies that a customized approach is appropriate, and one might imagine a protocol based on initial level and BMI, similar to starting insulin on a diabetic or levothyroxine in a patient with hypothyroidism, but no prospective studies that indicate how this should effectively be done. At the cost of $250 per serum of 25-hydroxyvitamin D concentration in our local healthcare system, there is a significant cost to using a treatment that has low likelihood of success, generating another round of treatment and a follow-up test.

Recently, the Endocrine Society published a clinical practice guideline regarding evaluation, treatment, and prevention of vitamin D deficiency (7). At the time our study was conducted, there was no such guideline, and, thus, the study was designed to evaluate the efficacy of the proposed standard treatment regimen by clinical experts. Given the fact that our study is retrospective, there are some inherent biases. Those practitioners who are checking vitamin D levels are more likely to be aggressive in treating vitamin D insufficiency or deficiency. Most of the patients were treated with ergocalciferol 50,000 IU for 8 weeks. This made further subgroup analysis difficult because of insufficient number of patients in the other treatment groups to be statistically significant. Also, a large portion of the initial patient population was excluded for various reasons as listed in the Materials and Methods section. This is in part related to the timeline of the study. At the onset of the Centricity EMR in the practice in 2005, there was not as much emphasis on obtaining vitamin D status as there is in today's practice environment. Also, we excluded patients if they did not have a repeated 25-hydroxyvitamin D concentration within 16 weeks; however, there is no guideline regarding the interval required to recheck 25-hydroxyvitamin D concentrations postrepletion. Additionally, there is a regional bias based on the fact that this was a single center study. Given the timeline of the study, markers of bone turnover, for example N-telopeptide testing, that are commercially available at this time, that could have shown a short-term change in bone health factors related to vitamin D repletion, were not, in fact, available to the authors at their institution and so were not evaluated. Finally, it was not the practitioner's standard practice to check calcium levels or screen for hypercalciuria when repleting with 8–12 weeks of ergocalciferol. The practitioners felt that the risk of toxicity was extremely low for this time period of replacement therapy, as supported by a recent systematic review, which concluded that the risk of hypercalcemia, hypercalciuria, or frank nephrolithiasis from repletion with doses up to 10,000 IU per day was not significantly greater than placebo groups (9).

## Conclusion

Pretreatment levels of 25-hydroxyvitamin D, BMI, season, and vitamin D dose are predictors of successful hypovitaminosis D treatment but are often ignored in deciding on a repletion regimen due to the ‘one size fits all’ approach previously recommended. Our data challenge this mantra and suggest that patients with initial 25-hydroxyvitamin D concentrations of <20 ng/mL, obesity, or evaluation during the winter should be treated with a higher total dose of ergocalciferol than currently recommended. A prospective, randomized trial is needed to determine the optimal treatment protocol accounting for these variables.
